# Impact of the internet on English language learning among university students: mediating role of academic self-efficacy

**DOI:** 10.3389/fpsyg.2023.1184185

**Published:** 2023-10-05

**Authors:** Ying Wang, Fakhra Yasmin, Ahsan Akbar

**Affiliations:** ^1^School of Foreign Studies, North China University of Water Resources and Electric Power, Henan, China; ^2^Department of Informatics and Quantitative Methods, Faculty of Informatics and Management, University of Hradec Králové, Hradec Králové, Czechia; ^3^International Business School, Guangzhou City University of Technology, Guangzhou, China

**Keywords:** students attitude toward internet use, academic self-efficacy, English language learning, Chinese universities, PLS-SEM

## Abstract

The internet is a valuable resource in a technologically evolved society. The extant literature suggests that their scientific and educational usages are still limited. The current study asserts that the internet can provide new learning environments and opportunities for Chinese university students, hence increasing their motivation to learn. Particularly, the current study considers this to be the case for learning a foreign language (English), which leads to more efficient and effective language learning experiences, as well as more positive attitudes toward the efficiency of the internet for educational purposes. Purposive and convenience sampling techniques were employed to gather data from 15 public and private Chinese universities (406 students), those who are currently enrolled in English language courses. The analysis was performed using partial least squares-structural equation modeling (PLS-SEM) on smart PLS 4 software. Results revealed that student’s attitude toward the use of internet positively and significantly influence English language learning. Moreover, the mediating variable academic self-efficacy positively and significantly mediates the relationship between students’ attitude toward use of internet and English language learning. The current study recommends that students’ academic self-efficacy in learning a new language can be enhanced by giving them opportunities to learn internet skills. Further, students’ confidence in their academic abilities can be boosted using student-centered teaching strategies.

## 1. Introduction

Today, people live in a global community where English has become the most widely spoken language worldwide (Ganapathy-Coleman, 2023). China has made remarkable progress in attracting foreign students and internationalizing its higher education system in recent years (Yasmin et al., 2021). As China is one of several countries where the teaching and learning of English is a priority due to the language’s widespread use (Yang and Salam, 2022; Wang and Diao, 2023). Besides that, Chinese education has struggled with higher student-teacher ratios, which hinder teacher-student contact and active learning (Huang et al., 2020). In this regard, language is a tool for sharing thoughts, ideas, and emotions with others (Tsai, 2022). However, it is believed that half of all people in the world can speak more than one language, and learning a second language has been linked to cognitive benefits at every stage of life. Moreover, English is the global language of communication, and a good grasp of it is the key to better education, jobs, and social status (Al Zoubi, 2018; Sung, 2022).

China is one of the leading economy which is using technological innovation to enhance productivity and efficiency in various industrial settings (He et al., 2023). The internet is one of the many resources available for learning English (Metruk, 2022) and can be thought of as a massive worldwide data network that links users in different countries. According to Huang et al. (2022), internet is a hub for global communication, enabling users to connect with one another and share ideas, information, and experiences through a variety of online communities and networks (Tao and Xu, 2022). Likewise, the internet is widely recognized as the most convenient resource for locating educational resources such as books and articles, etc. (Marhaditya, 2021). Academic institutions are looking for innovative and sustainable solutions in the rapidly changing environment (Chowdhury and Kabir, 2023; Pachot and Patissier, 2023). Sustainability practices in higher education institutions can also help in developing next generation of corporate leaders who make innovative and sustainable decisions (Yang et al., 2021). It is also becoming increasingly common for universities to use internet-based tools in their classrooms (Shang, 2022). For instance, Tsegay et al. (2022) stated that online feedback and discussion can improve teacher-student communication, class participation, learner motivation (Yang and Wu, 2022), and learning regulation (Huang et al., 2020).

Internet-based educational technology like learning management systems, social networking sites, and mobile learning can encourage student participation in lecture-based curriculum and develop critical thinking and problem-solving skills (Metruk, 2022). However, English language instruction has been given a technological boost by integrating the latest internet innovations into the classroom. Learning that takes place entirely online is called “fully online,” while “blended learning” blends in-person instruction with digital resources (Hysa et al., 2022). In other words, technology makes language learning environments more varied (Yang and Salam, 2022). Another element that contributes significantly to success in learning English is the academic self-efficacy which has a big impact on educational success. Likewise, learning styles of the students are also found to be significantly linked with their academic achievements (Yasmin et al., 2016a,b). According to Ran et al. (2022), an individual’s self-efficacy (SE) may be a value judgment conveyed through his or her self-perceptions. Learning, in his view, is affected by a person’s self-perception of how well he has done on a task, which in turn influences the learner’s progress (Kosimov, 2021). General academic SE is a student’s overall belief that they can handle the different academic challenges at university, and it is an important predictor of both happiness and academic performance (Nielsen et al., 2018). When students believe in their academic talents, they are less likely to be disheartened by failure, are less prone to delay, and put in more effort overall in their coursework (Richardson et al., 2012; Tossavainen et al., 2021). For example, prior research has revealed that one of the key elements in learning English is English self-efficacy. Because students will be learning on their own, their abilities must be considered when talking about using online resources to learn English (Gao, 2021; Tao and Xu, 2022).

University students struggle when they have to complete multiple assignments in different subjects. Thus, students might be more motivated to use technology in their learning if they thought it was easy (Liu et al., 2023). A person’s perception of a system’s usability can be determined by its perceived ease of use. In this regard, the perceived ease of using the internet for learning, includes Baidu and online teaching, as well as learning platforms (Tao and Gao, 2022). Using these methods, the importance of technology in the classroom might be highlighted. However, despite the potential benefits that technology may bring to students’ learning, they may forgo using it if they find it to be too time-consuming (Huang et al., 2022). Similarly, constant developments in internet technology are producing new types of learning environments that interest and inspire students.

The goal of the present study was to examine the effect of the internet on English language learning among Chinese students: the mediating role of academic self-efficacy. Today, quality-focused education has become the primary objective of education and the focus of English language study has shifted from classroom instruction to self-paced learning. The widespread use of Internet technology has given rise to a brand-new area of personal freedom, and the demand for English-language instruction continues to rise both domestically and internationally. In this regard, internet-based English instruction can be a powerful tool for maximizing the effectiveness and quality of instruction for individuals or groups (Shang, 2022). As learning English requires immersion in an English-speaking setting, but direct interaction with native speakers is not always possible (Islam, 2011). Therefore, English has become the international language of business, education, entertainment, and other fields. Furthermore, English language acquisition holds significant importance for numerous Chinese households, with a primary emphasis placed on instructing children in this particular linguistic domain. There is a consensus within the education community regarding the imperative for Chinese students to enhance their proficiency in the English language. In addition, there is limited research on the continuation of internet-based English language learning materials (Gao, 2021; Shang, 2022). This research will help elucidate the significance of internet technology for English language learning among Chinese students. Moreover, this research also highlights the importance of academic self-efficacy in English language learning.

## 2. Literature review

### 2.1. Theoretical framework (technology acceptance model)

The Technology Acceptance Model (TAM) is based on planned behavior. Davis et al. (1992) suggest that an individual’s motivation to do an action is a major factor in whether they do it. The definition of intentions serves to identify the factors that motivate behavior and they reveal the extent to which an individual is prepared to make an effort to do or perform a certain behavior (Ajzen, 1991). Perceived usefulness, perceived ease of use, and attitude toward technology are only a few of the variables that make up TAM and are responsible either directly or indirectly (Zhou et al., 2022), for explaining behavioral intentions concerning technology use (Liu et al., 2023). SE, subjective rules, and enabling conditions for technology use are some of the newer exogenous variables that have been incorporated into the model (Scherera et al., 2019). The theory of planned behavior states that there is a positive correlation between the strength of one’s desire to execute an action and the likelihood that the action will actually be carried out. The availability of opportunities and resources, for example, can greatly influence the likelihood that certain actions will occur (Ajzen, 1991). According to the model, the likelihood of a behavior being successful is contingent upon the presence of individual motivation, as well as the provision of both opportunity and support (Malureanu et al., 2021).

### 2.2. Impact of the internet on English language learning

Smart technology for daily life has become a social phenomenon as a result of the ongoing transformation of society. As a result of the rapid pace of societal change, people require lifelong occupational skills and studies have shown the importance of decision-making, problem-solving, and vocational abilities (Oh, 2020). Similarly, learning environment; virtual communities in an online English language learning forum could help learners motivate each other or give them a place to practice English with people who are interested in the same things they are (Tsegay et al., 2022). Besides that, hybrid e-learning helped university students get motivated and work together (Haider et al., 2021). In addition, online English instruction made shy students more willing to speak in front of a screen (Yang and Salam, 2022). For instance, a study conducted on university students’ attitudes toward using the internet for English learning in Taiwan by Huang et al. (2012) found that the perceived ease of use had a positive and significant effect.

Similarly, the internet is a highly helpful learning resource for people who are not natural English speakers (Huang et al., 2022). It can offer students convenient access to learning content that is available on demand and presented in text, speech, and video formats (Liu et al., 2023). Additionally, using internet-based resources allows one to overcome limitations imposed by time, distance, and geography while still learning from native speakers. Moreover, young people frequently use smartphones for communication, entertainment, and education (Gao, 2021; Metruk, 2022).

According to the principles of communicative language teaching, which focus on meaningful interaction, studies have shown that the internet can help people learn English (Tao and Gao, 2022). This demonstrates that the internet has a crucial effect on the development of communicative competence in the context of the English language. For this reason, students need to take the lead in their own English language education by actively engaging with a variety of learning materials (Lekawael, 2017; Zhang and Tananuraksakul, 2023). Thus, we hypothesized as:

H1: There is a significant effect of the internet on English Language Learning.

### 2.3. Internet and academic self-efficacy

The effectiveness of students in virtual learning environments is greatly influenced by internet and academic SE. For example, prior studies have mostly focused on how students perceive and assess technological tools for language learning, their adoption in classroom settings, and the variables affecting language learning effectiveness under technologically advanced conditions (An et al., 2021). Numerous internet-based educational tools have been implemented at different educational levels, facilitating communication between teachers and students and enabling knowledge sharing (Haider et al., 2022; Shang, 2022). Examples include Web-browsing tools and learning management systems (LMS) that allow teachers to upload instructional materials and post online assignments and questions (Huang et al., 2022).

A person’s SE in English is measured by how well they think they will be able to use English in a variety of situations, including speaking with others, listening to others, reading, and writing (Wang et al., 2018). Students who feel positive about learning have a better chance of quickly adapting to this new way of learning and tackling all the challenges they face. Students need a basic understanding of information technology so that they can feel comfortable and confident in their ability to learn and do well in online courses. Relevant SE constructs for online education include those related to both academic and internet literacy. When a person’s environment changes suddenly, it can affect their mental stability and how they act. Self-efficacy and psychological adaptation are useful predictors of student behavior in the virtual environment (Onu and Mbonu-Adigwe, 2021). Furthermore, flipped classrooms (FC) may also boost students’ SE due to better student-teacher interactions and more opportunities to watch peers master skills. Predictably, most FC studies on SE show positive results (Algarni and Lortie-Forgues, 2023). Consequently, the following hypothesis was formulated:

H2: There is a relationship between the internet and academic self-efficacy.

### 2.4. Academic self-efficacy and English language learning

Self-efficacy (SE) is defined as a person’s confidence in his or her ability to exert influence over his or her surroundings and achieve a desired outcome, as proposed by Bandura’s social cognitive theory (Bandura, 1993). Academic SE is a student’s confidence in his or her ability to learn and succeed (Lei et al., 2022). Evidence suggests that students with better levels of academic self-efficacy are more inclined to take on demanding assignments, whereas those with lower levels of SE are more likely to avoid those (Su et al., 2018).

Similarly, prior research supports the assumption that students with strong SE are more likely to speak a foreign language with confidence both within and outside of the classroom (Kosimov, 2021; Han et al., 2022). Wong (2005) discovered that learners’ SE has a substantial impact on the quality of their writing; students with high SE for writing demonstrate more writing progress than those with low SE for writing. In addition, studies acknowledge that students’ SE influences their overall learning and academic performance Ran et al., 2022). Researchers also found that SE beliefs affect motivation, learning, and academic success. Low SE beliefs can lead to avoidance and negative feelings, which can hurt performance and well-being as compared to better SE which helps in facing different challenges (Waddington, 2023). Learner SE research has shown that student self-efficacy beliefs affect foreign language learning motivation and achievement. Thus, language learners can develop self-regulation strategies and more positive and realistic attitudes toward foreign language learning by identifying low SE beliefs (Zhang, 2020). Thus, we developed the hypothesis as follows:

H3: There is a relationship between academic self-efficacy and English language learning.

### 2.5. Mediating role of academic self-efficacy between internet and English language learning

According to Alzubaidi et al. (2016), one of the most popular motivational theories is SE, which is termed as “beliefs in one’s capacity to organize and carry out the courses of action required to accomplish specific attainments” (Bandura, 1997). Academic self-efficacy, which refers to students’ assessments of their capacities to carry out learning activities, is a common word used to characterize SE in educational contexts (Honicke and Broadbent, 2016). It is typically seen as a crucial psychological component in students’ learning process (Han et al., 2021). Additionally, research indicates that student’s English proficiency and learning anxiety are mediated by students’ SE (Su et al., 2018). Moreover, students with better levels of SE are more likely to involve in behaviors that promote learning and success in the classroom (Hanham et al., 2021).

In existing research on students’ use of information and communication technologies for learning, SE is “students’ self-reliance in their ability to pick relevant technical solutions and effectively apply the selected technologies to accomplish learning objectives” (An et al., 2021). Students with a strong feeling of SE, as shown by Kim and Lorsbach (2005), are more likely to report high levels of oral competency and a feeling of confidence while speaking in public. Previous studies revealed that students’ SE in the English language positively affected their feedback decisions and behaviors in academic English course environments.

Baleghizadeh and Masoun (2013) explored that having a high sense of SE affected the speaking skills of students learning English. Those studying English as a second language saw an improvement in their communication abilities as a result. Numerous studies have shown that a student’s sense of competence has a significant bearing on how well they do in education. Additionally, English SE is a term that describes how confidently students believe they can learn English. Students’ SE in English refers to their faith in their abilities to carry on meaningful discussions, read and write in English, and understand spoken English (Wang et al., 2018). Also, research from the past shows that learning a language is linked to possible loss of face and humiliation, which affects students’ sense of SE. Numerous empirical studies have shown that one’s sense of competence is crucial to successful language acquisition. Stronger levels of SE in language learning are associated with greater levels of self-regulation (Bai and Wang, 2023). Based on above-mentioned literature following hypothesis was developed:

H4: Academic self-efficacy plays a mediating role in the association between the internet and English Language learning.

## 3. Research methodology

Students’ attitudes and motivations toward their online English-learning experiences are evaluated (Tsegay et al., 2022; Yang and Wu, 2022). The short-term attitude that arises from an appraisal of a student’s educational experience and occurs when the actual performance meets or surpasses the learner’s expectations (Abuhassna et al., 2020). Purposive and convenience sampling techniques (Etikan et al., 2016), were employed to gather data from 15 public and private university students, those who are currently enrolled in English language courses. The data was collected online through email and social media platforms (i.e., WeChat and Facebook) were used to distribute a link to the Google Doc survey. On the first page of the survey, all respondents were informed about anonymity, participation, and withdrawal policies. The data was gathered during 4 months, from November 2022 to February 2023. The data was collected at one time; hence the research is cross-sectional in nature. The researcher first obtains permission from their university’s boards and officials before gathering data for this study. To check for the CMV problem, Harman’s single factor test was applied in SPSS with all three variables (students’ attitude toward the uses of the Internet, academic self-efficacy, and English language learning) (Tehseen et al., 2017). The first factor had a variation of 19.66% in the findings. This is less than 50%, indicating the lack of CMV in the data.

G* power software suggests that 107 respondents are needed to get a power of 0.95 and a medium size effect size of 0.15 for this study (Kang, 2021). The researchers, however, collected information from 406 students, exceeding the required sample size. Researchers sent out 550 questionnaires and received responses from 438 students. After the removal of incorrect or missing data, 406 surveys were usable, yielding a response rate of 74%. This is a prevalent issue with cross-sectional data, in which a researcher obtains data through survey while targeting the same respondents. The response rate was quite encouraging in the difficult Covid-19 pandemic situation. The demographic information of the respondents was shown in Table 1. Out of the 406 students, 129 (31.8%) were female and 277 (68.2%) were male. The majorities of students are 18–23-year-old and have bachelor’s and master’s degree holders. There was 62.3% percent of participants from public universities, and just 37.7% were from private universities. When the same participants were employed to collect data for all the variables, common method variance (CMV) might be present in the results. CMV was present even though many procedural remedies were used, such as a cover letter to protect respondents’ anonymity, a definition of new terminology, short and basic questions, etc. (Tehseen et al., 2017). Furthermore, the impact of the CMV was investigated by correlating latent variables using the statistical method of Dziuban and Shirkey (1974) “Correlation Matrix Process” (CMP). Since the major variables correlated less than 0.90, CMV was not found using this method. Furthermore, a comprehensive collinearity assessment strategy was used to check at CMV.

**Table 1:** Descriptive statistics.

Demographics	Categories	Frequency	Percent
Gender	Female	129	31.8
	Male	277	68.2
Age	18–20	146	36.0
	21–23	179	44.1
	24–28	75	18.5
	>28	6	1.5
Education	Bachelor	121	29.8
	Masters	192	47.3
	MS	80	19.7
	PhD	13	3.2
University	Public university	253	62.3
	Private university	153	37.7

### 3.1. Measures

All construct items were derived from previously published works, and data was gathered through questionnaires. Before conducting a complete study analysis, researchers performed a pilot study to verify the reliability of the measuring instrument (Van Teijlingen and Hundley, 2001). All responses were measured on a five-point Likert scale, from “strongly disagree” (1) to “strongly agree” (5). This tool is ideal for data gathering since it facilitates the simple and quick acquisition of quantitative data. In this study, students’ attitude toward the use of Internet was used as an independent variable based on 17-items scales developed by Hunjra et al. (2010). Furthermore, the mediating variables academic self-efficacy scale measured the extent to which the students believed that they were confident in mastering lessons and skills taught in their English classes. This 5-scale was adapted from Midgley et al. (2000). Lastly, for dependent variables the 16-items scale was used to measure English language learning, developed by Önal et al. (2022).

## 4. Research findings

**Figure 1:**
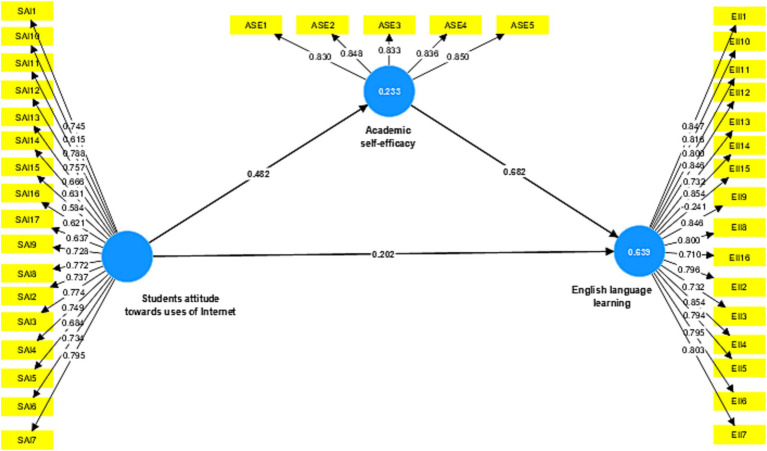
Measurement model assessment.

Subsequently, correlations were performed using SmartPLS software version 4 (Hair et al., 2021), to examine the relationship between students’ attitudes toward uses of the Internet and English language learning. A mediating role of academic self-efficacy on the relationship between students’ attitude toward uses of the Internet and English language learning was examined using a bootstrapping approach with 5,000 sub-samples and a mediation analysis provided using partial least squares-structural equation modeling (PLS-SEM) (Sarstedt et al., 2022). One of the advantages of PLS-SEM is that it may be modified to meet specific needs while also providing better statistical power (Hair et al., 2017). In the next steps of the PLS-SEM statistical analysis, the measurement and structural models were assessed separately. Before using the information to calculate relationships between variables, it is necessary to ensure that the measurement model meets all of the necessary criteria for validity and reliability (see Figure 1).

### 4.1. Measurement model

The indicator of reliability reveals item loadings range from −0.241 to 0.854, as seen in Table 2 demonstrating convergent validity. Factor loadings in the range of 0.40 and 0.70 should be eliminated only if doing so increases the average variance extracted (AVE), as stated by Hair et al. (2017). Yet, if the average variance extracted is larger than 0.5, Cronbach’s Alpha (α) is greater than 0.7, and the composite reliability (CR) is greater than 0.7, the convergent validity of the concept is considered acceptable (Hair et al., 2021). Table 2 and Figure 1 presented that all composite reliability is over 0.80, indicating all three constructs; academic self-efficacy, students’ attitude toward the uses of Internet, and English language learning are acceptable measurements of their respective constructs.

**Table 2:** Factor loadings, AVE, and CR.

Items	Factor loading	α	CR	AVE	Items	Factor loading	α	CR	AVE
English language learning		0.945	0.957	0.608	Students’ attitude toward uses of the Internet		0.938	0.945	0.504
EII1	0.847				SAI1	0.745			
EII2	0.796				SAI2	0.737			
EII3	0.732				SAI3	0.774			
EII4	0.854				SAI4	0.749			
EII5	0.794				SAI5	0.684			
EII6	0.795				SAI6	0.734			
EII7	0.803				SAI7	0.795			
EII8	0.800				SAI8	0.772			
EII9	0.846				SAI9	0.728			
EII10	0.816				SAI10	0.615			
EII11	0.800				SAI11	0.788			
EII12	0.846				SAI12	0.757			
EII13	0.732				SAI13	0.666			
EII14	0.854				SAI14	0.631			
EII15	−0.241				SAI15	0.584			
EII16	0.710				SAI16	0.621			
Academic self-efficacy		0.895	0.923	0.704	SAI17	0.637			
ASE1	0.830								
ASE2	0.848								
ASE3	0.833								
ASE4	0.836								
ASE5	0.850								
									

α, Cronbach’s alpha; CR, composite reliability; AVE, average variance extracted.

### 4.2. Discriminant validity

To evaluate the consistency of discrimination, researchers used the Heterotrait-Monotratit (HTMT) method proposed by Henseler et al. (2015). The threshold value was initially calculated using HTMT. If the test result is over the HTMT’s minimum threshold for non-discrimination, the existence of bias is established. When the correlation is close to one, it is unclear what the appropriate HTMT cutoff value should be. At first, HTMT was used to establish an appropriate cutoff value. As compared to the HTMT cutoff, higher results indicate that discrimination does not occur. When the correlation is close to one, the precise HTMT threshold value is unclear. Some lenient researchers have proposed a value of 0.90, while others have supported 0.85 (Hair et al., 2021). Second, the discriminant validity was evaluated and established by analyzing HTMT values with a confidence interval of less than one. Second, the discriminant validity was evaluated and established by analyzing HTMT values with a confidence interval of less than one. When 1 is eliminated from the interval range, the constructs become empirically clear. As demonstrated in Table 3, the HTMT values between the constructs are less than 0.85. Hence, discriminating validity is acknowledged in this study.

**Table 3:** Discriminant validity through Heterotrait-Monotrait ratio (HTMT).

Variables	Academic self-efficacy	English language learning	Students’ attitude toward uses of the Internet
Academic self-efficacy			
English language learning	0.735		
Students’ attitude toward uses of the Internet	0.514	0.554	

### 4.3. Structural model

The next stage, after confirming that the measurement model is appropriate, is to analyze the structural model (Sarstedt et al., 2022). Each hypothesis is allocated a logical relationship in a structural model, and the hypothesized relationship is often assessed using a path coefficient. Including the coefficient of determination (R^2^), impact size (f^2^), and predictive significance (Q^2^) Cohen (1988). Generally, the t value of a coefficient is used to establish its statistical significance. In two-sided testing, the t-values 1.65 (significance level = 10%), 1.96 (significance level = 5%), and 2.57 (significance level = 1%) are the standard critical values. In this study, the hypothesis was confirmed if the hypothesis *t*-value (relationship between variables) was greater than the threshold value of 1.96. When the *t*-value of the hypothesis (relationship between variables) falls below 1.96, the hypothesis is considered invalid.

**Figure 2:**
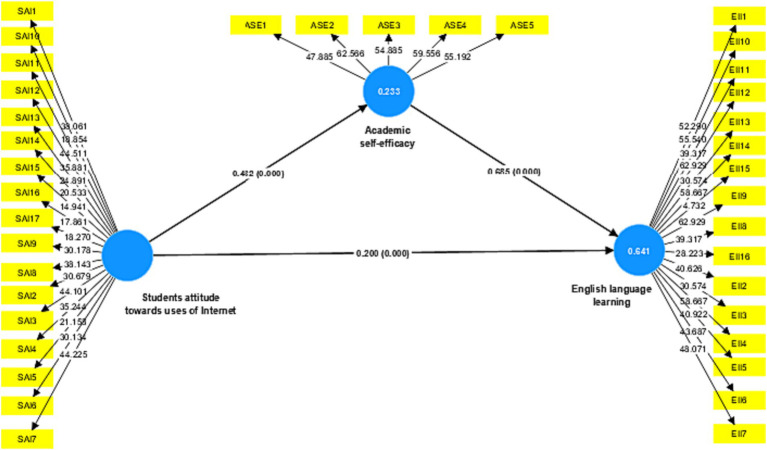
PLS-path analysis of (*n* = 5,000 bootstrapped samples).

Figure 2 presented that Students’ attitude toward the uses of the Internet has a positive and significant influence on English language learning (*β* = 0.200, *p* < 0.001) also *t*-value is equal to 4.958 > 1.96. The value of β demonstrates the percentage change, illustrating that a one-unit change in SAI results 0.200 unit change in English language learning. The results reveal that around 20% of the change in the dependent variable English language learning is seen, and a value of *p* < 0.001 implies a higher degree of significance, giving strong grounds for accepting hypothesis H1. Furthermore, the direct effect of SAI on ASE (*β* = 0.482, *t* = 11.065, *p* < 0.001) and ASE on English language learning (*β* = 0.685, *t* = 20.009, *p* < 0.001) were also positive and significant. Hence, all three direct hypotheses H1, H2, and H3 were accepted.

The predictive power of a model is measured by its R^2^ Coefficient of Determination. Its value, which may vary from zero to one, indicates the total influence of exogenous latent factors on underlying latent variables. Higher *R*^2^-values reflected more robust explanatory power. Cohen (1988) defines high, moderate, and low *R*^2^-values as 0.75, 0.50, and 0.25, respectively. Table 4 illustrates the value of R^2^ for Academic self-efficacy was 0.233, which is considered high and indicated that Students attitudes toward uses of the Internet explained 23.3% of the variance on ASE. Moreover, the English language learning R^2^ value was 0.639, which indicates that Students’ attitudes toward use of Internet and ASE can predict a 63.9 percent change in English language learning. The model has high prediction accuracy, as seen in Figure 2. Q^2^ (predictive relevance) is used to determine how significant the internal model is regarding developing predictions. D was determined to be 7, whereas Q^2^ was determined using the blindfold method. Predictive significance was calculated using a cross-validated redundancy method. Predictive significance is shown when the value is larger than zero, and the model is not predictively relevant when it is less than zero (Cohen, 1988). Table 5 shows the model’s predictive relevance for Academic self-efficacy (Q^2^ = 0.194) and English language acquisition (Q^2^ = 0.543) of endogenous components.

**Table 4:** Structured-equation-model results.

Hypotheses	Relationship among constructs	*β*	Sample mean	S.D.	*T*-values	F square	*p*-values	LLCI 2.5%	ULCI 97.5%	Remarks
	Direct effect									
H1	Students’ attitude toward uses of the Internet - > English language learning	0.200	0.202	0.040	4.958	0.087	0.000	0.127	0.283	Supported
H2	Students’ attitude toward uses of the Internet - > Academic self-efficacy	0.482	0.485	0.044	11.065	0.303	0.000	0.396	0.567	Supported
H3	Academic self-efficacy - > English language learning	0.685	0.684	0.034	20.009	0.990	0.000	0.614	0.746	Supported
	Indirect effect (mediation analysis)									
H4	Students’ attitude toward uses of the Internet - > Academic self-efficacy - > English language learning	0.331	0.332	0.032	10.274		0.000	0.270	0.396	Supported

**Table 5:** Determining coefficient for the partial least squares method.

Variables	R-square	R-square adjusted	Q^2^ predict
Academic self-efficacy	0.233	0.231	0.196
English language learning	0.639	0.637	0.543

The degree to which the value of R^2^ changes when a given exogenous construct is removed from a model to ascertain whether or not the exclusion has an impact on the endogenous constructs is a measure of the effect size (f^2^). Cutoff values of 0.02 for a small effect size (f^2^), 0.15 for a medium size (f^2^), and 0.35 for big size (f) was proposed by Hair et al. (2021). Table 4 shows that Students’ attitude toward the use of the Internet has a small influence on English language learning (0.087), whereas the medium effect on Academic self-efficacy (0.303). However, the mediating variable ASE has a large effect on English language learning (0.990). Finally, the model also predicted and confirmed that Academic self-efficacy would act as a mediator on the relationship between Students’ attitude toward use of Internet and English language learning. Table 4 shows that the indirect effect of SAI on English language learning through mediator ASE is positive and significant (*β* = 0.331, *t* = 10.274, LLCI = 0.270, ULCI = 0.396). The Lower Limit Confidence Interval (LLC) and Upper Limit Confidence Interval (ULCI) values are without having any zero between both limits, which clarifies that the results are significant. Therefore, hypothesis H4 was also accepted.

## 5. Discussion

The goal of the current study was to examine the effect of the internet on English language learning among Chinese students: the mediating role of academic self-efficacy. Technology is becoming more and more popular as a way to bridge formal and informal settings in target language learning and give students the chance to use technology keenly and efficiently both inside and outside of the classroom (An et al., 2021). One of the primary objectives of employing contemporary technology is to actively involve students in the process of language acquisition, thereby fostering practical and realistic development of English language skills. Most countries around the world where English is not the native language have emphasized teaching English as a means of fostering vital communication skills for the twenty-first century classroom (Bai et al., 2022; Tao and Gao, 2022). In this regard, the internet is widely regarded as one of the most valuable technological advancements of contemporary society, providing significant benefits not only in our personal lives but also in our professional endeavors. A growing number of studies and scholarly journals dedicated to the field of education have focused on the impact of the internet on higher education. Moreover, internet has a significant influence on improving and streamlining the English-learning process (Khalaf, 2018).

Following were the hypotheses of the present study. First, it was hypothesized that there is a significant impact of the internet on English Language Learning. This study’s results were supported by other studies that have linked students’ internet attitudes to their motivation and interest in Internet-based learning (Peng et al., 2006; Yang and Wu, 2022). While earlier research has shown that incorporating technology into language classes improves student performance, relatively few studies have examined the role of affective qualities like SE in domains as narrow as language acquisition (Namaziandost and Çakmak, 2020). According to the results of a recent study, learning a new language and increasing one’s language skills are both facilitated by internet use. Previous research has shown that incorporating internet use into the classroom increases students’ motivation to study English and promotes student responsibility. Internet use in education enhances learning, teaching, and communication (Ganiyevna, 2023). Similarly, several researchers have pointed out that the internet encourages students to practice their foreign languages in authentic settings (Jamalifar and Chalak, 2014). Moreover, technology in the second language writing classroom makes learners independent, is an excellent way to teach foreign cultures and language, and generally improves students’ attitudes. According to several studies of language learners, the internet contains authentic and real language in a meaningful context, and by observing this material, learners become language creators rather than passive recipients (Xayriniso, 2023).

Second, it was hypothesized that there is an association between the internet and academic SE. The findings of the current study were in line with the literature. Research reveals that students’ perceptions of the ease with which they can use the internet for learning (particularly Baidu and online LMS) are related to their confidence in their ability to use these resources effectively for educational purposes (Huang et al., 2022; Shang, 2022). Likewise, studies have shown that when students have high levels of SE, they are more engaged in their online courses, and they take more responsibility for their learning. Students with high online learning SE are more interested in technology and learning challenges and have lower anxiety during online learning (Hong et al., 2023).

Third, it was hypothesized that there is a relationship between academic SE and English language learning. The current study’s findings were consistent with prior research. Researchers have found that students with better SE levels have a good chance of succeeding at language learning than those with low levels (Ran et al., 2022). Similarly, previous research has demonstrated that students who have a high sense of SE in language acquisition tend to have more successful outcomes (Kosimov, 2021). In addition, numerous empirical studies have demonstrated that academic SE plays a crucial role in language acquisition. Bai and Wang (2023) found that language learners with higher SE beliefs are more self-regulated. Academic self-efficacy and interest are crucial to English language learning. As a key motivational variable, interest can result in increased SE and self-regulation (Bai et al., 2022).

Lastly, it was hypothesized that academic SE plays a mediating role in the association between the internet and English Language learning. The present study results were in line with prior literature. High SE makes students more interested in learning tasks and improves their performance, which in turn raises their SE. On the other hand, having a low sense of SE leads to poor results and lowers performance across a variety of tasks (Kuo and Belland, 2019). According to prior study, students’ internet self-efficacy is a measure of how confident they are in their ability to succeed in online interactions that happen on information and communication technology systems and platforms (Zhou et al., 2022). Other studies on online learning and SE showed that SE is crucial to student success. These studies show that high SE increases academic achievement, student engagement, and self-confidence (Bi et al., 2023). A study found that students with high SE employed a wider variety of learning strategies to enhance their speaking skills. Similarly, SE was found to be a strong predictor of motivation in language learning. Furthermore, recent research has established a correlation between academic SE and public speaking performance (Kusuma and Waluyo, 2023).

## 6. Conclusion

The study aimed to find the influence of the internet on English language learning among Chinese students by considering the mediating role of academic self-efficacy. The hypotheses of the present study were found to be supported. The study’s findings led to the conclusion that the internet has a notable influence on the process of learning the English language and on academic self-efficacy. Furthermore, a noteworthy correlation was identified between academic self-efficacy and the acquisition of English language skills. Moreover, a hypothesis was formulated suggesting that academic self-efficacy (SE) acts as a mediator in the relationship between internet usage and English language learning.

Based on the study findings, it is concluded that the student’s confidence in their abilities is a key factor in their success in improving their English skills. It is interesting to see how SE can motivate a learner to work hard and get good results. To boost the confidence of struggling students, teachers should encourage them to try and provide frequent, targeted feedback for their replies throughout the course. SE can be boosted through classroom collaboration with peers, the selection of manageable learning objectives, and the acceptance of constructive criticism from teachers. Tasks designed to encourage students to take charge of their education shall be used to achieve this goal. Individuals will enhance and fortify their capacity to autonomously acquire English language skills through the utilization of the internet, thereby cultivating the aptitude for continuous learning, ultimately benefiting both themselves and society at large. Moreover, technological advancements have been present in various academic disciplines, including the field of language education. English teachers should embrace technological advancements and effectively utilize them as a means to enhance their teaching and learning methodologies.

## 7. Theoretical implications

The internet helps teachers and a student find lots of materials, enriches teachers’ pedagogy when choosing materials and methods for English learning, and engages students in creating a new English learning experience. According to the findings of the aforementioned literature, using the internet as part of an English language learning environment is seen as a motivating tool for the language learner as it facilitates the acquisition of language proficiency in the contexts of both communication and daily life. The research found that the Technology acceptance model influenced students’ intentions to use online learning applications. By incorporating e-learning into university courses, managers and developers of e-learning must help students improve their positive perception and enhance their English language skills in the four areas of speaking, listening, reading, and writing (Shahid et al., 2023).

## 8. Practical implications

To begin with, it is imperative for instructors of English as a second language to foster a sense of self-assurance among their students by actively promoting the development of personalized learning strategies. In order to enhance the efficacy of online language-learning systems, developers must employ novel technologies that provide continuous and prompt feedback for students’ learning progress. Second, the process of creating educational resources should include instructor encouragement of student self-assessment. Third, the internet is a tool for classrooms because it facilitates the sharing of resources, allows teachers to better tailor their pedagogy to the needs of their students, and encourages students to actively participate in the development of innovative approaches to learning English. Fourth, students’ internet self-efficacy can be improved by giving them places to learn internet skills. Fifth, students’ confidence in their academic abilities can be boosted through the use of student-centered teaching strategies. The instructor should observe the personality attributes of students to determine the type of virtual learning environment that will maximize the achievement of each student.

It is imperative that educators be provided with enhanced opportunities for professional development and family education through collaborations with colleges and schools. The inclusion of topics pertaining to SE and motivational theories should be incorporated within these programs. In addition, methods should be presented to foster a sense of intrinsic worth, SE, and a growth mindset among students. In the context of SE, teachers can place an emphasis on skill building that boosts students’ competence through real-world mastery experience. Teachers need effective methods of instruction if they want their students to succeed in the English language. Additionally, instructors should complement students’ progress. Furthermore, teachers can promote SE in a way that is similar to how they can help students develop a growth mindset. They can do this by recognizing efforts and improvements, limiting social comparison, and helping students set their internal standards for judging results.

## 9. Limitations and future directions

Listed below are some limitations of the study. First, the cross-sectional design of this study meant that the data could be collected at a single point in time. Second, as the internet becomes increasingly used as a learning tool, it is vital to conduct longitudinal research to determine whether students’ views on the utility of such tools for education will shift. Third, future studies need to focus on the misuse of the internet by young students. It is a significant issue that impacts both students and parents (Tao and Xu, 2022). Fourth, students’ perspectives on internet learning may also be influenced by factors like (class level, learning technique, and motivation type), which suggest the need for more study. In order to enhance the efficacy of online education, it would be beneficial to investigate the potential extension of the findings presented in this study to encompass educators and institutions of higher learning. This will facilitate the continued advancement of this significant research domain.

## Data availability statement

The raw data supporting the conclusions of this article will be made available by the authors, without undue reservation.

## Author contributions

All authors listed have made a substantial, direct, and intellectual contribution to the work and approved it for publication.
